# The Effect of Anti-Inflammatory Dimethylmalonic Acid on the Neurobehavioral Phenotype of a Neonatal ASD Model Induced by Antiepileptic Valproic Acid

**DOI:** 10.3390/biomedicines13071765

**Published:** 2025-07-18

**Authors:** Xiuwen Zhou, Xiaowen Xu, Lili Li, Yiming Jin, Qing Wang, Xinxin Wang, Meifang Jin, Hong Ni

**Affiliations:** Department of Brain Science, Institute of Pediatric Research, Children’s Hospital of Soochow University, Suzhou 215025, China

**Keywords:** dimethylmalonic acid, valproic acid, autism spectrum disorder, neonate, neurobehavior

## Abstract

**Background**: Valproic acid (VPA) is a medication used to treat epilepsy, bipolar disorder, and migraine. If taken during pregnancy, it can cause neural tube defects (NTDs) and leads to offspring ASD behavioral phenotype. It has recently been found that early postnatal VPA exposure can also induce the ASD phenotype, but the details of model production and intervention still need further investigation. Dimethylmalonic acid (DMM), a competitive inhibitor of succinate dehydrogenase, blocks the key element succinate of OXPHOS, decreasing the secretion of anti-inflammatory cytokines and ROS production. However, it is still unclear whether DMM is involved in the repair of developmental brain injuries. **Objectives**: The aim of this study was to evaluate the intervention effect and optimal dosage of DMM on behavioral phenotypes using a neonatal mouse VPA autism model. **Methods**: This experiment consists of two parts. The first part observed the effects of different concentrations of VPA on the development and neurobehavioral phenotype of mice. The second part determined the intervention effect of DMM on a developmental VPA autism model and determined the optimal therapeutic dose. **Results**: We found that the 40 mg/mL concentration had a greater impact on the neural reflex damage in mice. Moreover, DMM treatment can partially improve the neurobehavioral damage in the VPA model, and 20 mg/kg has the best intervention effect. **Conclusions**: This study provides valuable model construction data for further exploring the mechanism of DMM treatment for an ASD phenotype induced by VPA exposure in neonates.

## 1. Introduction

Autism spectrum disorder (ASD) is a heterogeneous neurodevelopmental disorder characterized by social communication disorders, repetitive and abnormal sensory motor behaviors, and narrow ranges of interest or activity [1]. ASD can last a lifetime and seriously affects the quality of life of patients, causing huge economic losses and social burdens to families and society. The treatment of ASD is a clinical priority, with education and rehabilitation currently being the main focus. Recent randomized controlled trials have confirmed that methods such as sensory integration training [2], music therapy [3], and cognitive behavioral therapy [4] have certain therapeutic effects on ASD. In China, in addition to the advantages of traditional Chinese medicine acupuncture and moxibustion in treating ASD [5], electromagnetic stimulation [6,7], parent–child therapy [8,9], virtual reality intervention technology [10], and other methods have also been used to treat ASD. However, there is relatively little research on drugs for treating autism in clinical practice, and there is still a lack of drugs targeting core symptoms. Therefore, it is urgent to explore new treatment methods from the perspective of mechanisms.

Although the etiology of ASD has not been fully elucidated, the influence of environmental factors is widely recognized. The main environmental factors include the following: (1) exposure to drugs during pregnancy, such as valproic acid (VPA) and selective serotonin reuptake inhibitors; (2) Exposure to toxic substances during pregnancy, such as heavy metals, insecticides, etc.; (3) Viral and bacterial infections during pregnancy; (4) Lack of vitamins and trace elements, etc. [11,12]. Valproic acid (VPA) is a medication used to treat epilepsy, bipolar disorder, and migraine. If taken during pregnancy, it can expose developing embryos to VPA, causing neural tube defects (NTDs). One mechanism by which VPA may lead to NTD is the interruption of cellular signaling through oxidative stress [13]. Exposure to VPA in the developing brain increases intracellular reactive oxygen species (ROS) production and oxidative stress damage, and induces autism-like behavioral disorders (social, repetitive, stereotyped, and sensorimotor) [14]. However, current research on oxidative stress mechanisms is still relatively preliminary and lacks ideal intervention drugs.

The selective accumulation of succinic acid, an intermediate product in the citric acid cycle, is a common metabolic characteristic in ischemic tissues during oxidative stress. During ischemia, the citric acid cycle is reversed, and succinate dehydrogenase SDH converts fumarate into succinate. Dimethylmalonic acid (DMM), as a competitive inhibitor of succinate dehydrogenase, reduces the SDH-mediated accumulation of succinate, blocks the key element of oxidative phosphorylation, succinate, and reduces the secretion of anti-inflammatory cytokines [15]. Recent preclinical studies have shown that in a pig ischemia–reperfusion model, dimethyl succinate can reduce the accumulation of succinic acid, thereby reducing the production of reactive oxygen species and protecting endothelial glycocalyx [16]. DMM can also alleviate brain damage in a rat model of cardiac arrest by inhibiting SDH [17]. In animal models of ischemic stroke in vivo and oxygen and glucose deprivation/reoxygenation in vitro, we have recently demonstrated that DMM can prevent ischemic brain injury [18]. However, it is still unclear whether DMM is involved in the repair of developmental brain injuries.

Therefore, this study attempts for the first time to explore the intervention effect and optimal dosage of DMM on a behavioral phenotype using a neonatal mouse VPA autism model in order to lay the foundation for subsequent mechanism research.

## 2. Materials and Methods

### 2.1. Quality Control of Animals During Transportation and Among Tests

Postnatal day 14 (P14) *C57BL/6J male mice* were purchased from Suzhou Xinuosai Biotechnology Co., Ltd., Room 307, Building 2, Maishanlong Mansion, No. 168, Yuxin Road, Suzhou Industrial Park, Suzhou, China. The mice were housed in an environment with a temperature of 25 °C, relative humidity of 50–70%, and a 12 h light/dark cycle.

Stress-reducing protocol during transportation: The animals were provided and transported by a company located in the same city as our hospital. Each litter of mice is individually packaged and transported in breathable cardboard boxes during transportation. The cushioning material inside the cardboard box contains larger wood chips. Previous studies have shown that mice prefer manipulable large particle bedding, which can effectively reduce their stress levels [19]. In addition, the following pressure relief measures were taken during transportation: 1. Temperature: Maintain at 20–26 °C to avoid excessive temperature differences (with a maximum change of 3 °C per hour) and prevent thermal stress. 2. Ventilation and oxygen supply: The transport container has sufficient ventilation holes (with an area not less than 10% of the container surface area). 3. Lighting: Avoid direct sunlight and maintain a soft transportation environment.

Stress-reducing protocol among tests: The laboratory feeding and testing process can also generate stress stimuli [20], for which we have taken the following measures: 1. Mother mice were housed together with their respective offspring in a cage. The cage for raising mice adopts a brown transparent cage, which can meet the mice’s preference for low light intensity well [21]. 2. The cages were regularly cleaned by professional staff, who washed their hands and changed their lab coats during the cleaning process, effectively preventing odor transmission and reducing stress in mice [22]. 3. Through adopting an intelligent independent ventilation cage, the cage was purchased from Suzhou Suhang Technology Co., Ltd. (Suzhou, China). The temperature, humidity, pressure, and air exchange rate inside the cage were monitored in real-time. 4. The animals were still nursing at the time of purchase and were not weaned for research purposes, as a litter of mice consists of mother and offspring. The young mice were able to eat freely. After 1–2 h of arrival at the animal room, we began to manipulate the animals. We only administered the drugs to the young mice through intraperitoneal injection, which has minimal stimulation and does not affect breastfeeding. In the practical operation, we could also observe the breastfeeding situation of mother mice to offspring mice. It could be seen that the nest mice were in a relatively comfortable environment with less stress.

Randomized allocation of animal litters: There was one female mouse and 6–8 offspring mice in each cage. A total of 1–2 offspring mice (labeled with gentian violet) were randomly allocated from each cage to each experimental group. In this way, the offspring of each experimental group were randomly selected from different cages, so the behavioral outcomes were caused by drug intervention rather than littering, ensuring drug effect behavior rather than specific littering behavior.

### 2.2. Establishment of the VPA Mouse Model

The production of VPA mouse model and animal gender selection were based on the research of Zhou et al. [23]. Previous studies on autistic-like behavior induced by VPA have shown that gender has different effects on behavioral phenotypes. Schneider et al. (2008) found that in the offspring of rats treated by prenatal exposure to VPA, males exhibited increased anxiety, increased repetitive stereotyped behavior, and decreased social behavior, while the treated females only showed an increase in repetitive movements [24]. Regarding the ASD model induced by taking VPA in the early postnatal period, Ornoy et al. found that males exhibited more severe ASD-like behaviors and higher overall autism scores than females. Specifically, males exhibit greater impairments in social novelty preferences, frequency of grooming, and cognitive rigidity (T-maze), indicating stronger autism-like behavior in males [25]. Accordingly, in the present study, fourteen-day-old neonatal C57BL/6J male mice weighing (6.5–7.7) g were selected, with the first day after birth defined as P0. On P14, the autism model group received a single intraperitoneal injection of 400 mg/kg valproic acid (VPA), while the control group was administered an equivalent volume of saline via intraperitoneal injection.

### 2.3. DMM Administration Method

P14 C57BL/6 mice received an intraperitoneal injection of VPA. Half an hour after the VPA injection, the mice were intraperitoneally injected with dimethyl malonate (DMM) at doses of 5, 10, 20, and 40 mg/kg, purchased from Sigma-Aldrich (St. Louis, MO, USA, Cat#136441-250G). DMM was intraperitoneally injected once, respectively, at P15, P16, and P17 [16]. Since dimethyl malonate (DMM) is insoluble in water, DMSO is required to assist in dissolution. The DMM formulation parameters are summarized in Table 1.

### 2.4. Experimental Design

The experimental design of this study consists of two parts.

**Figure 1:**
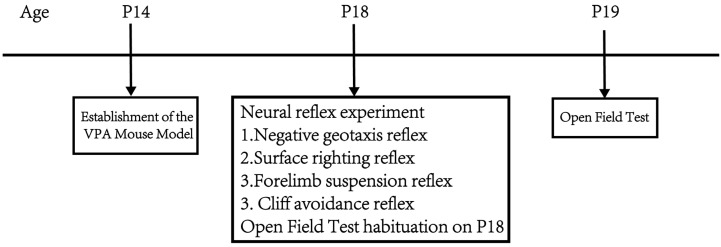
Flowchart of the first part of the experimental design.

Part 1: Observe the effects of different concentrations of VPA on mouse body weight, neurodevelopment, exploratory ability, and spontaneous activity. At P14, mice were given a single intraperitoneal injection of 400 mg/kg VPA to establish the VPA-induced autism mouse model. The mice were divided into a sham surgery group, a 400 mg/kg VPA (injection concentration 40 mg/mL) group, and a 400 mg/kg VPA (injection concentration 20 mg/mL) group. n = 10 per group, as the literature [26] indicates that n = 10 is sufficient for meaningful statistical interpretation of behavioral experiments. From P14 onwards, daily records of mouse body weight changes were maintained. At P18, negative geotaxis, cliff avoidance, forelimb suspension, and surface righting tests were conducted. The mice were placed in the open field at p18 and allowed to freely explore for 5 min to facilitate their adaptation to the experimental environment. The open field test was conducted at P19. Figure 1 shows the flow chart of the first part of the experimental design.

**Figure 2:**
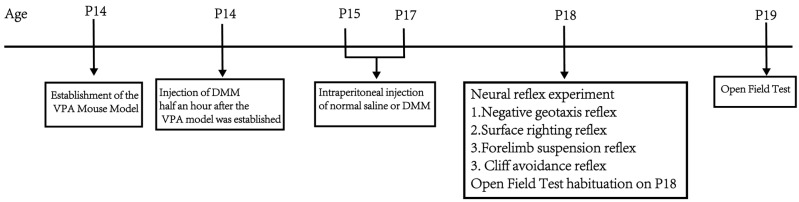
Flowchart of the second part of the experimental design.

Part 2: Determine the intervention effect of dimethyl malonate (DMM) on the developmental VPA autism model in terms of body weight, neurodevelopment, exploratory ability, and spontaneous activity, as well as the optimal therapeutic dose. The VPA autism model was established by intraperitoneal injection of 400 mg/kg VPA in mice at P14. Half an hour after the VPA model, DMM was injected intraperitoneally (5, 10, 20, and 40 mg/kg). The mice were divided into a sham operation group, VPA group, VPA + 5 mg/kgDMM treatment group, VPA + 10 mg/kgDMM treatment group, VPA + 20 mg/kgDMM treatment group, and VPA + 40 mg/kgDMM treatment group; n = 10/group, and the weight changes in mice were recorded daily from P14. Negative geotaxis, cliff avoidance, forelimb suspension, and surface righting tests were performed at P18. The mice were placed in the open field at P18 and allowed to freely explore for 5 min to facilitate their adaptation to the experimental environment. The open field test was conducted at P19. Figure 2 shows the flowchart of the second part of the experimental design.

### 2.5. Body Weight Measurement

Body weight monitoring was performed using an electronic scale (Bailing, Suzhou, China) to record the body weights of mice in each group, aiming to assess their physical development on postnatal day 14, postnatal day 15, postnatal day 17, and postnatal day 18. After weighing each mouse, the scale was reset to zero to ensure accurate and error-free results.

### 2.6. Negative Geotaxis Reflex [27]

The negative geotaxis reflex is used to observe the vestibular and proprioceptive functions of mice. First, a plastic board was prepared, inclined at a 45-degree angle. The experiment involves placing the mouse head-down at the center of the plastic board. Normally, the mouse will adjust its posture to head-up. The time taken for the mouse to complete this postural adjustment was recorded.

### 2.7. Cliff Avoidance Reflex [28]

The cliff avoidance reflex is used to assess the reaction ability of mice. The experiment involves positioning the mouse’s forepaws and nose near the edge of the experimental platform. The time from placement at the edge until the mouse turns more than 90 degrees was recorded.

### 2.8. Forelimb Suspension Reflex [29]

The forelimb suspension test evaluates the grip strength and reactivity of the mouse’s forepaws. First, a metal rod (approximately 0.5 cm in diameter) was fixed at a height of 50 cm above the ground. At the start of the experiment, the mouse is allowed to grasp the metal rod with its forepaws. The duration for which the mouse maintains its grip on the rod was recorded.

### 2.9. Surface Righting Reflex [30]

The surface righting reflex is used to assess the coordination ability of mice. The experiment begins by placing the mouse in a supine position on the experimental platform. Normally, the mouse will quickly right itself to a prone position. The time taken for the mouse to fully right itself is recorded.

### 2.10. Open Field Test [31]

The open field test is used to evaluate the exploratory ability and spontaneous activity of mice. The experiment is conducted in an acrylic glass box measuring 72 cm in length, 72 cm in width, and 50 cm in height, with the bottom evenly divided into 16 small squares. The central four squares are defined as the central zone, while the remaining areas are considered the peripheral zone. Each mouse is gently placed in the center of the glass box and allowed to freely explore for 5 min to acclimate to the environment. After 24 h, each mouse is again gently placed in the center of the glass box and allowed to freely explore for 5 min. The total distance traveled, movement speed, and the number of entries into the central zone are recorded.

## 3. Results

### 3.1. Part One: Comparison of Two VPA Concentrations

#### 3.1.1. Reduced Body Weight and Increased Mortality in Mice After VPA Modeling

The first day after birth was defined as P0. On P14, the autism VPA model group received a single intraperitoneal injection of 400 mg/kg VPA (n = 10), and changes in body weight were recorded starting from P14. The control group received an equivalent dose of normal saline (NS) via intraperitoneal injection. For the 20 mg/mL VPA group, two mice died at P16 and P18, respectively, resulting in a final survival count of eight. For the 40 mg/mL VPA group, three mice died at P15, P16, and P17, respectively, resulting in a final survival count of seven.

**Figure 3:**
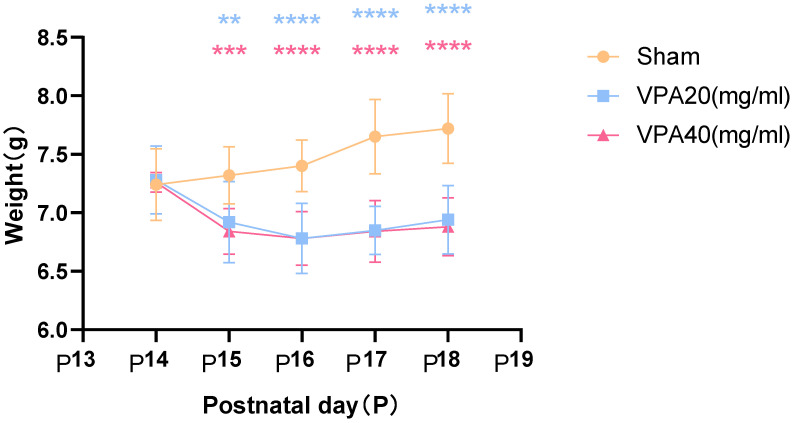
Changes in body weight of mice in each group were statistically compared using two-way ANOVA. All data are expressed as mean ± standard deviation. ** indicates VPA20 (mg/mL) vs. saline (NS) group, *p* < 0.01. *** indicates VPA40 (mg/mL) vs. saline (sham) group, *p* < 0.001. **** indicates VPA20 (mg/mL) vs. saline (sham) group, *p* < 0.0001. **** indicates VPA40 (mg/mL) vs. saline (NS) group, *p* < 0.0001, n = 10. VPA: valproic acid-induced autism model; sham: saline control.

The body weight of mice in the VPA model group showed a significant decrease starting from the second day after modeling, as shown in Figure 3, with statistically significant differences. However, there was no statistically significant difference in body weight changes between mice treated with 20 mg/mL VPA and those treated with 40 mg/mL VPA. The above results indicate that 400 mg/kg VPA-induced injury leads to a significant decrease in mouse body weight, and the extent of the decrease is independent of VPA concentration.

#### 3.1.2. Impaired Neurodevelopment in VPA-Treated Mice

**Figure 4:**
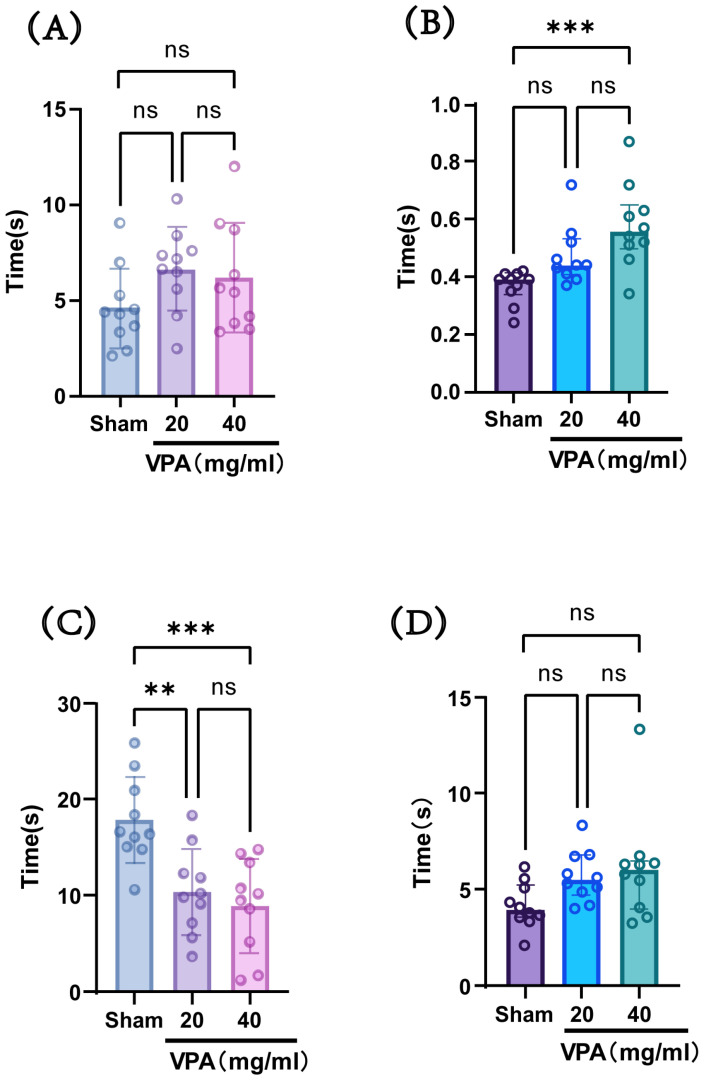
(**A**) Negative geotaxis reflex statistical chart; (**B**) surface righting reflex statistical chart; (**C**) forelimb suspension reflex statistical chart; (**D**) cliff avoidance reflex statistical chart. Data normality was assessed using the Shapiro–Wilk test. Results indicated that datasets (**A**,**C**) followed a normal distribution; therefore, one-way ANOVA was employed for statistical comparison, with data expressed as mean ± standard deviation (SD). The F value for dataset (**A**) is 1.967, and the F value for (**C**) is 10.61. Datasets (**B**,**D**) violated normality assumptions and were analyzed using the Kruskal–Wallis test, with results presented as median ± interquartile range (IQR). ns: Not statistically significant, ** *p* < 0.01, *** *p* < 0.001; n = 10. VPA: valproic acid-induced autism model; sham: saline control.

The VPA model was established in P14 mice with a dose of 400 mg/kg, and behavioral tests were conducted at P18 to evaluate neurodevelopmental indices across groups. As shown in Figure 4, compared with the saline-treated group (sham group), the model group (VPA group) exhibited a statistically significant increase in the time required for surface righting and a decrease in forelimb suspension time. However, there were no statistically significant differences observed in negative geotaxis reflex or cliff avoidance reflex times. These results indicate that although the two concentrations showed no statistically significant differences in their effects on the aforementioned indices, the 40 mg/mL concentration led to longer surface righting times and shorter forelimb suspension times compared with the 20 mg/mL concentration, suggesting a greater impairment of neural reflexes at the higher concentration. Considering the advantage that the injection volume of the 40 mg/mL solution was half that of the 20 mg/mL solution, we selected a VPA dose of 400 mg/kg (40 mg/mL concentration) for subsequent studies.

#### 3.1.3. Reduced Exploratory Ability and Spontaneous Activity in VPA Mice

**Figure 5:**
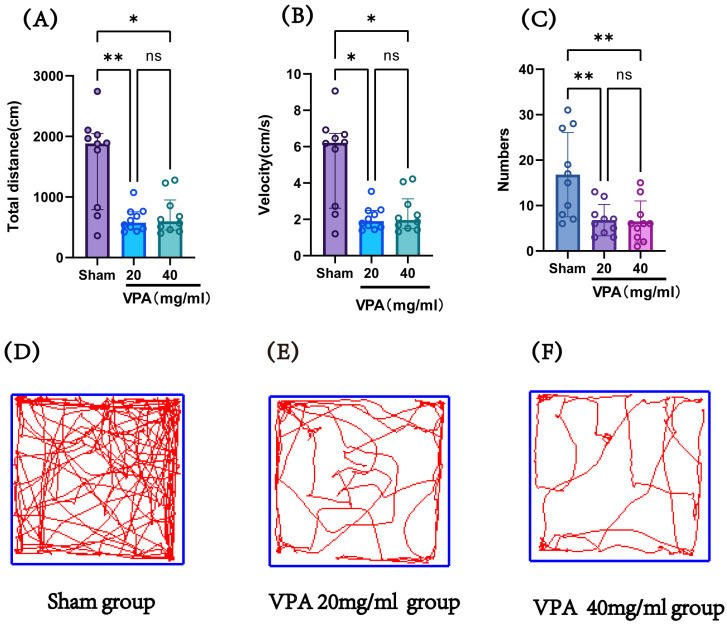
(**A**) The total distance traveled by each group of mice in the open field test, (**B**) the speed of mice in each group in the open field test, (**C**) the number of times each group of mice entered the central grid in the open field test, (**D**–**F**) trajectory maps of the open field test for the sham group, VPA 400 mg/kg at 20 mg/mL concentration group, and VPA 400 mg/kg at 40 mg/mL concentration group. Normality was assessed using the Shapiro–Wilk test. The results indicated that datasets (**A**,**B**) violated normality assumptions and were analyzed using the Kruskal–Wallis test, with results presented as median ± interquartile range (IQR). Dataset (**C**) followed a normal distribution; therefore, one-way ANOVA was employed for statistical comparison, with data expressed as mean ± standard deviation (SD). The F value for dataset (**C**) is 8.706. ns: Not statistically significant, * *p* < 0.05, ** *p* < 0.01; n = 10. VPA: valproic acid-induced autism model; sham: saline control.

The VPA model (400 mg/kg) was established in P14 mice, and an open field test was conducted on P19 to record the total distance traveled and speed of the mice, evaluating their exploratory ability and spontaneous activity. Figure 5 shows the statistical results of the open field test for the saline control group (sham), 400 mg/kg VPA (40 mg/mL concentration) mice, and 400 mg/kg VPA (20 mg/mL concentration) mice. As illustrated in Figure 5, the VPA model mice exhibited a significantly reduced total distance traveled, movement speed, and number of entries into the central zone compared to the saline control group (sham). The differences were statistically significant, whereas no statistically significant differences were observed between the two VPA concentration groups. These results indicate that VPA-induced damage leads to reduced exploratory ability and spontaneous activity in neonatal mice, promoting the onset of autism. Furthermore, this impairment was independent of VPA concentration.

### 3.2. Determine the Intervention Effects of Dimethyl Malonate (DMM) on Body Weight, Neurodevelopment, Exploratory Ability, and Spontaneous Activity in the Developmental VPA Autism Model, as Well as the Optimal Therapeutic Dosage

#### 3.2.1. The DMM Treatment Group Ameliorated the VPA-Induced Reduction in Mouse Body Weight and Decreased Mouse Mortality

The first day after birth was defined as P0. At P14, the autism VPA model group received a single intraperitoneal injection of 400 mg/kg VPA at a concentration of 40 mg/mL (n = 10). Half an hour later, intraperitoneal injections of DMM (5, 10, 20, and 40 mg/kg) were administered, respectively, for three consecutive days. Changes in mouse body weight were recorded starting from P14. In the 5 mg/kg DMM group, one mouse died at P15 and another at P16, resulting in a final survival count of eight. In the 10 mg/kg DMM group, one mouse died at P15, leaving nine surviving mice. All mice in the 20 mg/kg DMM group survived, while in the 40 mg/kg DMM group, one mouse died at P15 and another at P16, resulting in a final survival count of eight.

**Figure 6:**
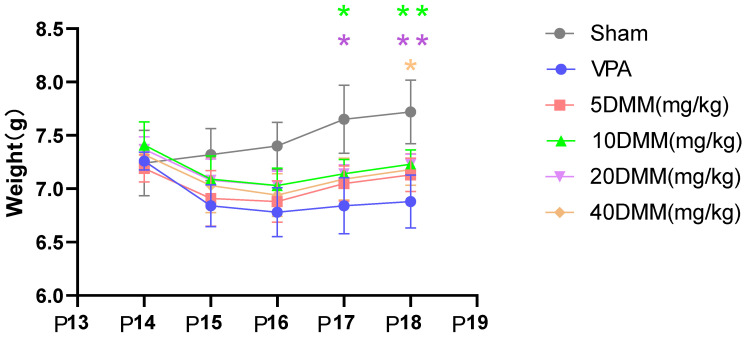
The changes in body weight of mice in each group were statistically compared using two-way ANOVA. All data were expressed as mean ± standard deviation. * indicates p17 10 mg/mL DMM treatment group vs. VPA model group, *p* < 0.05; * indicates p17 20 mg/mL DMM treatment group vs. VPA model group, *p* < 0.05; ** indicates p18 10 mg/mL DMM treatment group vs. VPA model group, *p* < 0.01; ** indicates p18 20 mg/mL DMM treatment group vs. VPA model group, *p* < 0.01; * indicates p18 40 mg/mL DMM treatment group vs. VPA model group, *p* < 0.05. n = 10. VPA: valproic acid-induced autism model; sham: saline control; DMM: dimethyl malonate.

Starting from P17, body weight differences were observed between the 20 mg/kg and 10 mg/kg mouse groups and the VPA model mouse group, as shown in Figure 6. The body weights of the 20 mg/kg and 10 mg/kg mouse groups were significantly higher than those of the VPA model group, with statistically significant differences (*p* < 0.05). At P18, the body weights of the 20 mg/kg and 10 mg/kg DMM mice remained significantly higher than those of the VPA model group, showing statistically significant differences (*p* < 0.01). The body weight of the 40 mg/kg mouse group was also significantly higher than that of the VPA model group (*p* < 0.05). These results indicate that DMM treatment can reduce the mortality rate of VPA mice, with the highest survival rate observed in VPA mice treated with 20 mg/kg DMM. Additionally, DMM treatment can mitigate the weight loss induced by VPA.

#### 3.2.2. The DMM Treatment Group Ameliorated Partial Neurodevelopmental Impairments Induced by VPA in Mice

**Figure 7:**
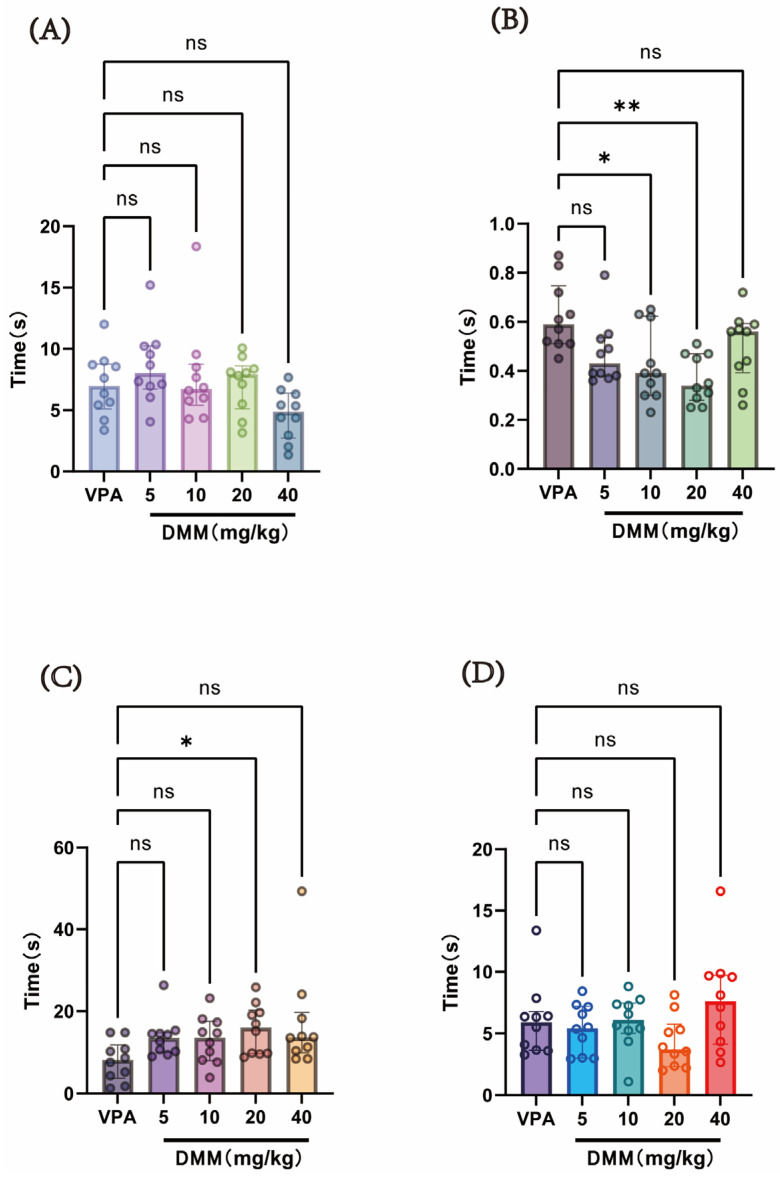
(**A**) Negative geotaxis reflex statistical chart; (**B**) surface righting reflex statistical chart; (**C**) forelimb suspension reflex statistical chart; (**D**) cliff avoidance reflex statistical chart. Data normality was assessed using the Shapiro–Wilk test. The results indicated that the data were not normally distributed; therefore, the Kruskal–Wallis test was employed for statistical comparison, with data presented as median ± interquartile range (IQR). ns: Not statistically significant, * *p* < 0.05, ** *p* < 0.01, n = 10. VPA: valproic acid-induced autism model; DMM: dimethyl malonate.

At P14, a single intraperitoneal injection of 400 mg/kg VPA (concentration: 40 mg/mL) was administered to the autism VPA model group (n = 10). Half an hour later, intraperitoneal injections of DMM (5, 10, 20, and 40 mg/kg) were given, with continuous administration for 3 days. Behavioral tests were conducted at P18 to evaluate neurodevelopmental indices across groups. As shown in Figure 7, compared with the VPA model group, the DMM treatment groups significantly reduced the time required for surface righting in mice, with statistically significant differences. The 20 mg/kg DMM treatment group exhibited the most pronounced effect. Furthermore, mice treated with 20 mg/kg DMM exhibited significantly prolonged forelimb suspension time. However, no statistically significant differences were observed between the DMM treatment groups and the VPA model group in negative geotaxis, cliff avoidance, or the time required for these tasks. These results indicate that DMM treatment can partially improve neurodevelopmental impairments caused by VPA.

#### 3.2.3. The DMM Treatment Group Ameliorated the VPA-Induced Reduction in Exploratory Ability and Spontaneous Activity in Mice, Improving Autism-like Symptoms in VPA-Exposed Mice

**Figure 8:**
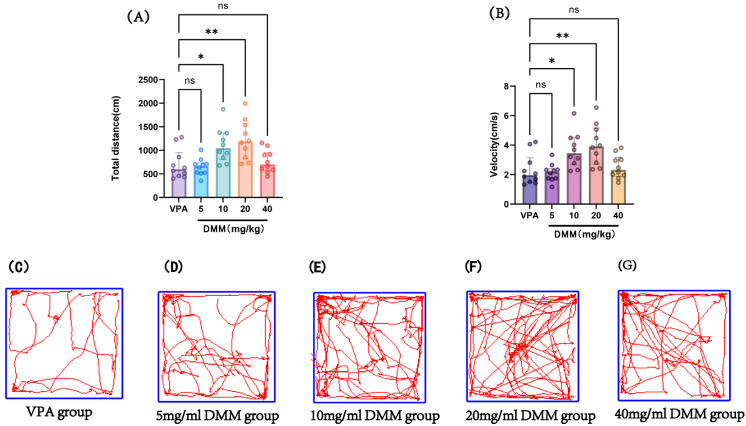
(**A**) Total distance in the open field test, (**B**) velocity in the open field test, (**C**–**G**) movement trajectories of mice in the open field test for the VPA group, 5 mg/mL DMM group, 10 mg/mL DMM group, 20 mg/mL DMM group, and 40 mg/mL DMM group, respectively. Data normality was assessed using the Shapiro–Wilk test. The results indicated that the data were not normally distributed; therefore, the Kruskal–Wallis test was employed for statistical comparison, with data presented as median ± interquartile range (IQR). ns: Not statistically significant, * *p* < 0.05, ** *p* < 0.01, n = 10. VPA: valproic acid-induced autism model; DMM: dimethyl malonate.

At P14, the autism VPA model group received a single intraperitoneal injection of 400 mg/kg VPA at a concentration of 40 mg/mL (n = 10). Half an hour later, mice were intraperitoneally injected with DMM at doses of 5, 10, 20, and 40 mg/kg, respectively, for three consecutive days. On P18, adaptation was performed, followed by an open field test at P19 to record the movement distance and speed of mice in each group, as shown in Figure 8. Compared with the VPA model group, the 10 mg/kg and 20 mg/kg DMM treatment groups exhibited significantly increased movement distance and speed, with statistically significant differences. The 20 mg/kg DMM treatment group showed the best performance. This indicates that DMM treatment can ameliorate the reduced exploratory ability and spontaneous activity in VPA mice, improving their autism-like symptoms.

The above results indicate that DMM treatment can partially improve the neurobehavioral damage in the VPA model, and 20 mg/kg has the best intervention.

## 4. Discussion

The first part of this study explored for the first time the effects of two different concentrations (40 mg/mL and 20 mg/mL) of VPA at the same dose (400 mg/kg) on mouse body weight, survival rate, neurological development, exploratory ability, and spontaneous activity. The results showed that although there was no statistically significant difference in the effects of the two concentrations on the above detection indicators, compared with 20 mg/mL, the 40 mg/mL concentration had a longer surface righting time and a shorter vertical suspension time, indicating that the 40 mg/mL concentration had a greater impact on the neural reflex damage in mice.

The prenatal exposure to antiepileptic drugs and toxic teratogen VPA is a classic autism environment mouse model [32]. Later, it was found that early postnatal exposure to VPA could also induce ASD behavioral phenotypes, thereby extending the time span of VPA-induced ASD [25]. In 2006, Wagner et al. induced ASD-like behavior in mice by injecting VPA on the 14th day after birth (postnatal day 14, P14) [33], which is equivalent to the late developmental stage of the third trimester of the human fetal brain [34]. The advantage of this rodent’s postnatal model is to avoid possible interference with maternal metabolism and the possibility to track the sequence of behavioral changes caused by VPA [25]. Strangely, newborn exposure to VPA has a completely opposite effect on the survival of brain neurons. Yochum et al. found that BALB/c mice injected on P14 with 400 mg/kg VPA had up to a 10-fold increase in TUNEL-positive cells in the dentate gyrus of the hippocampus [35]. However, in the experimental model of neonatal hypoxic–ischemic brain injury conducted by Kabakus et al., 5-day VPA treatment had certain protective and therapeutic effects on neuronal apoptosis in both hemispheres [36]. Given that these studies only detected pathological phenotypes, it is possible that the inherent limitations of pathological indicators have led to the contradictory results mentioned above. Therefore, it is necessary to explore changes in neurobehavioral indicators in vivo in order to improve the stability of detection variables and the reliability of results.

In this study, we followed the method of Furnari et al. [27] and administered a single intraperitoneal injection of 400 mg/kg VPA to newborn C57BL/6J male mice on postnatal day 14 (P14). However, Furnari’s research and recent studies by Zhou et al. using the same VPA model, dosage, and administration time have not explored the effects of different VPA concentrations on test indicators while maintaining the same VPA dosage. In fact, while maintaining the same dosage, different drug concentrations mean different liquid injection volumes, with higher concentrations resulting in lower injection volumes, which may affect test results. For example, Sudakov et al. compared the anxiolytic, psychostimulant, and analgesic effects of various volumes of ethanol solution in different concentrations, but with the same dose. The experiment compared the effects of different volumes (40, 15.5, and 5 mL/kg) of ethanol (dose 2 g/kg) on anxiety levels, motor activity, and pain sensitivity in rats. The results showed that injecting animals with 40 mL/kg volume significantly increased the time spent on the arm opening in the elevated plus maze. On the other hand, however, administration in a volume of 5 or 15.5 mL/kg had little effect on the level of anxiety [37]. Based on the above findings and considering that the animals in our current study have a relatively light body weight (average 7.2 g) at P14, which limits their tolerance to intraperitoneal injection, we are concerned that injecting too much fluid may affect growth, development, and survival. To address the above concerns, in this study, we compared the effects of two different injection concentrations (20 mg/mL, 40 mg/mL) of the same dose of VPA (400 mg/kg) on the body weight and neurobehavior. We found no statistical difference in survival rate and body weight between the two concentrations, indicating no significant difference in their impact on growth and development. In terms of neurobehavior, compared with 20 mg/mL, the 40 mg/mL concentration has a longer surface righting time and a shorter forelimb suspension time, indicating that 40 mg/mL concentration has better modeling effect. Considering the advantage of reducing the liquid volume by half when injected at 40 mg/mL compared to 20 mg/mL, we chose a VPA dose of 400 mg/kg and a concentration of 40 mg/mL in the second part of the study.

The second part of this study explored the intervention effects of DMM on the body weight and neurobehavior of VPA model mice, as well as the optimal therapeutic dose of DMM. The dose–effect experiment of DMM includes four doses (5, 10, 20, and 40 mg/kg). We followed the method of Zhou et al. and selected neurobehavioral parameters as effect evaluation indicators [23]. We found that VPA mice treated with 20 mg/kg DMM had the highest survival rate. Compared with the VPA model group, the DMM treatment group significantly reduced the surface righting time, and the 20 mg/kg DMM treatment group had the most significant effect (Figure 7B). Furthermore, mice treated with 20 mg/kg DMM exhibited significantly prolonged forelimb suspension time (Figure 7C). In addition, in the open field test, compared with the VPA model group, the 10 mg/kg and 20 mg/kg DMM treatment groups showed significant improvements in exercise distance and speed, with the 20 mg/kg group being more pronounced (Figure 8A,B). The above results indicate that DMM treatment can partially improve the neurobehavioral damage in the VPA model, and 20 mg/kg has the best intervention effect.

Interestingly, our DMM intervention has a similar effect to Ligustride used by Zhou et al. [23]. It should be noted that the VPA model we used, animal age, and the neurobehavioral detection indicators used for efficacy evaluation are completely consistent with Zhou et al.’s study, indicating that DMM and Ligustride improve early neurobehavioral damage in the VPA model through the same mechanism. In Zhou’s study, Ligustride treatment inhibited autistic-like phenotypes via a ferritinophagy-dependent pathway. Meanwhile, in the rat models of pentylenetetrazole (PTZ)-induced epileptogenesis and kainic acid (KA)-induced status epilepticus (SE), DMM significantly reduced oxidative stress and iron levels, alleviating the severity of epileptic seizures and neuronal damage [38]. We recently also found that DMM significantly suppressed neuronal ferroptosis and improves cell survival under OGD/R (oxygen and glucose deprivation/reoxygenation) conditions in vitro [18]. Therefore, it is reasonable to speculate that the protective effect of DMM on neurobehavioral damage caused by early postnatal VPA exposure is achieved by inhibiting signaling pathways related to neuronal ferroptosis, which merits further research.

In conclusion, this study provides valuable model building data for future research on the phenotype of ASD induced by VPA exposure in newborns through DMM intervention.

## Figures and Tables

**Table 1:** DMM formulation preparation table.

Dosage	DMM Stock Solution	DMSO	Processing Steps
5 mg/kgDMM	4.4 μL	50 μL	1. Add DMM stock to DMSO 2. Vortex mix thoroughly 3. Dilute with saline to 10 mL
10 mg/kgDMM	8.8 μL	50 μL	
20 mg/kgDMM	17.6 μL	50 μL	
40 mg/kgDMM	35.2 μL	50 μL	

## Data Availability

The original contributions presented in this study are included in the article. Further inquiries can be directed to the corresponding author.
